# Supplementary material for: Striatal adenosine A2A receptor neurons control active-period sleep via parvalbumin neurons in external globus pallidus
Source: eLife. 2017 Oct 12;6:e29055. doi: 10.7554/eLife.29055 (PMC5655138; doi:10.7554/eLife.29055)
Supplement: Transparent reporting form [file elife-29055-transrepform.docx]

***eLife’s* transparent reporting form**

We encourage authors to provide detailed information *within their submission* to facilitate the interpretation and replication of experiments. If you have any questions, please contact us: [editorial@elifesciences.org](mailto:editorial@elifesciences.org).

**Sample-size estimation**

- You should state whether an appropriate sample size was computed when the study was being designed
- You should state the statistical method of sample size computation and any required assumptions
- If no explicit power analysis was used, you should describe how you decided what sample (replicate) size (number) to use

Please outline where this information can be found within the submission (e.g., page numbers or figure legends), or explain why this information doesn’t apply to your submission:

An appropriate sample size of our study was estimated when the study was being designed in accordance with previous published articles of our and other sleep research groups (Qu WM, et al, J Neurosci; Chen L, et al, Neuropsychopharmacology; Anaclet C, et al, Nat Neurosci; Han Yong, et al, Curr Biol; Min Xu, et al, Nat Neurosci).

**Replicates**

- You should report how often each experiment was performed
- You should include a definition of biological versus technical replication
- The data obtained should be provided and sufficient information should be provided to indicate the number of independent biological and/or technical replicates
- If you encountered any outliers, you should describe how these were handled
- Criteria for exclusion/inclusion of data should be clearly stated
- High-throughput sequence data should be uploaded before submission, with a private link for reviewers provided (these are available from both GEO and ArrayExpress)

Please outline where this information can be found within the submission (e.g., page numbers or figure legends), or explain why this information doesn’t apply to your submission:

1. The biological replication was employed in our study. We defined that the biological replication was measured the effect in several different mice of same strain.

2. Only two mice power spectrum data were removed and described in Page 27 Lines 591-593 to read “Two mice power spectrum data were removed, as there was no NREM and REM sleep following vehicle or CNO injection over 3h or 4h (Figure 1H; Figure 6H,6I).”

**Statistical reporting**

- Statistical analysis methods should be described and justified
- Raw data should be presented in figures whenever informative to do so (typically when N per group is less than 10)
- For each experiment, you should identify the statistical tests used, exact values of N, definitions of center, methods of multiple test correction, and dispersion and precision measures (e.g., mean, median, SD, SEM, confidence intervals; and, for the major substantive results, a measure of effect size (e.g., Pearson's r, Cohen's d)
- Report exact p-values wherever possible alongside the summary statistics and 95% confidence intervals. These should be reported for all key questions and not only when the p-value is less than 0.05.

1. Statistical analysis methods were described in Page 34-35.
2. Raw data for each histogram was presented in figure by the scatter plot (Fig 1D, 1H-1K; Fig 1-fig supplement 2B; Fig 1-fig supplement 3E-3H; Fig 1-fig supplement 4A-4H; Fig 1-fig supplement 5E, 5J; Fig 2L; Fig 3E, 3J, 3M-3O; Fig 3-fig supplement 2E, 2J; Fig 4G, 4H; Fig 5G, 5J; Fig 6G-6I; Fig 6-fig supplement 1B, 1C).
3. Exact values of N for each experiment were described in figure legends of all figures and all supplementary figures.
4. The p-value of each experiment was described in figure legends of all figures and all supplementary figures.

Please outline where this information can be found within the submission (e.g., page numbers or figure legends), or explain why this information doesn’t apply to your submission:

(For large datasets, or papers with a very large number of statistical tests, you may upload a single table file with tests, Ns, etc., with reference to page numbers in the manuscript.)

**Additional data files (“source data”)**

- We encourage you to upload relevant additional data files, such as numerical data that are represented as a graph in a figure, or as a summary table
- Where provided, these should be in the most useful format, and they can be uploaded as “Source data” files linked to a main figure or table
- Include model definition files including the full list of parameters used
- Include code used for data analysis (e.g., R, MatLab)
- Avoid stating that data files are “available upon request”

Please indicate the figures or tables for which source data files have been provided:

1. Numerical data in Figure 1 were described in Figure 1**–**source data 1.
2. Numerical data in Figure 1**–**figure supplement 2 were described in Figure 1**–**figure supplement 2**–**source data 1.
3. Numerical data in Figure 1**–**figure supplement 3 were described in Figure 1**–**figure supplement 3**–**source data 1.
4. Numerical data in Figure 1**–**figure supplement 4 were described in Figure 1**–**figure supplement 4**–**source data 1.
5. Numerical data in Figure 1**–**figure supplement 5 were described in Figure 1**–**figure supplement 5**–**source data 1.
6. Numerical data in Figure 2 were described in Figure 2**–**source data 1.
7. Numerical data in Figure 3 were described in Figure 3**–**source data 1.
8. Numerical data in Figure 3**–**figure supplement 2 were described in Figure 3**–**figure supplement 2**–**source data 1.
9. Numerical data in Figure 4 were described in Figure 4**–**source data 1.
10. Numerical data in Figure 5 were described in Figure 5**–**source data 1.
11. Numerical data in Figure 6 were described in Figure 6**–**source data 1.
12. Numerical data in Figure 6**–**figure supplement 1 were described in Figure 6**–**figure supplement 1**–**source data 1.
